# A Novel Marine Mammal *Coxiella burnetii*—Genome Sequencing Identifies a New Genotype with Potential Virulence

**DOI:** 10.3390/pathogens12070893

**Published:** 2023-06-29

**Authors:** Brett R. Gardner, Nathan L. Bachmann, Adam Polkinghorne, Jasmin Hufschmid, Mythili Tadepalli, Marc Marenda, Stephen Graves, John P. Y. Arnould, John Stenos

**Affiliations:** 1 Melbourne Veterinary School, The University of Melbourne, Werribee, VIC 3030, Australia; huj@unimelb.edu.au (J.H.); mmarenda@unimelb.edu.au (M.M.); 2 Major Mitchell Consulting, Buderim, QLD 4556, Australia; nathan.bachmann@agrf.org.au (N.L.B.); adam@majormitchellconsulting.com.au (A.P.); 3 Australian Rickettsial Reference Laboratory, University Hospital Geelong, Geelong, VIC 3220, Australia; mythili.tadepalli@barwonhealth.org.au (M.T.); graves.rickettsia@gmail.com (S.G.); john.stenos@barwonhealth.org.au (J.S.); 4 School of Life and Environmental Sciences, Deakin University, Burwood, VIC 3125, Australia; john.arnould@deakin.edu.au

**Keywords:** IS1111, multiple-locus variable-number tandem-repeat analysis (MLVA), abortion, marine mammal, south-eastern Australia, Australian fur seal, *Arctocephalus pusillus doriferus*

## Abstract

The obligate intracellular bacterial pathogen *Coxiella burnetii* has been identified in a few species of marine mammals, some of which are showing population declines. It has been hypothesized that *C. burnetii* in marine mammals is a distinct genotype that varies significantly from the typical terrestrial genotypes. It appears to lack an IS1111. Isolates originating from Australian marine animals have a distinctly non-Australian profile of multiple-locus variable-number tandem-repeat analysis (MLVA). Extracted *Coxiella* DNA of Australian fur seal placental origin was sequenced using the Novaseq platform. Illumina 150 bp paired-end reads were filtered and trimmed with Trimgalore. The microbial community present in the sequenced genome was evaluated with Kraken and Bracken software using the NCBI database. A phylogenetic analysis was performed using 1131 core genes. Core genes were identified using Panaroo and inputted into Iqtree to determine the maximum-likelihood tree. A second phylogenetic tree was created using *Rickettsiella grylii* and using seven housekeeping genes. Results were compared with the *C. burnetii* Nine Mile RSA439 virulent genome. This new Australian marine mammal isolate of *Coxiella* (PG457) appears to be a novel genotype that lacks IS1111 and has a distinct MLVA signature (ms26, ms27, ms28, ms30, and ms31). The presence of genes for multiple virulence factors appears to give this genotype sufficient pathogenicity for it to be considered a possible causative agent of abortion in Australian fur seals as well as a potential zoonotic risk.

## 1. Introduction

*Coxiella burnetii* is the intracellular, Gram-negative, bacterial causative agent of Q fever. It has adapted exceptionally well to an obligate intracellular existence within the normally inhospitable host-protective phagolysosome [1]. The lack of genetic diversity across all known *C. burnetii* genotypes is considered evidence of this pathogen only recently emerging from one of the typical *Coxiella*-like endosymbionts of ticks [2]. Q fever is known to cause disease in both humans and other mammals [3]. Ruminants, especially goats and cattle, are considered some of the most important reservoirs of this zoonotic pathogen, especially in Australia [4,5]. Historically considered predominantly a disease of workers in the livestock industry, especially those working in abattoirs, it has recently become a disease concern in wildlife rehabilitators [5,6]. *C. burnetii* is also considered an emerging disease in wildlife, where it has been shown to cause reproductive failure in multiple species [7]. There is a considerable paucity of data in wildlife globally and within Australia [5,7]. Concerns over the impact of *C. burnetii* in wildlife have recently extended to marine mammals with the detection of this pathogen in several species of pinnipeds, some of which are undergoing population declines [8,9]. Beyond the conservation impacts, the presence of this pathogen in wildlife is also of interest given the potential for wildlife to act as reservoirs of *C. burnetii* infection in humans and domesticated animals [10].

Most genotypes that have been isolated from wildlife appear to cluster into genomic groups I–III, based predominantly on studies from Europe [10]. Despite *C. burnetii* being detected in more than 109 wild mammal species, there is still a significant lack of sequencing data to investigate the importance of this pathogen in wildlife and the zoonotic spillover risk [7]. It has been hypothesized that wildlife may harbor genetic variations that could allow for the emergence of further pathogenic variations of the organism [10]. Within an Australian context, the macropods appear to include some of the best-known potential reservoir species [11,12]. Most studies in Australian wildlife have focused on serology and molecular prevalence. There do not appear to be any sequencing data available for *C. burnetii* in terrestrial Australian wild mammals, and to the best of the authors’ knowledge, no genotyping or sequencing data are available for *C. burnetii* from any marine mammals.

It has nevertheless been hypothesized that *C. burnetii* in marine mammals is a unique genotype with key features such as the absence of IS1111 (insertion sequence 1111) [13,14]. A recent isolate of *C. burnetii* from Australian fur seals (*Arctocephalus pusillus doriferus*) was analyzed using multiple-locus variable-number tandem-repeat analysis (MLVA) for three loci critical to the Australian genotypes (ms24, ms28, and ms33) [15], but only ms28 was amplified [14]. At present, it is unknown whether the *C. burnetii* identified in northern fur seals (*Callorhinus ursinus*), Steller sea lions (*Eumetopias jubatus*), Pacific harbor seals (*Phoca vitulina richardsi*), harbor porpoises (*Phocoena phocoena*), and Australian fur seals is the same organism between all the marine mammals [8,9,16]. Nor is it currently known whether it is a definitive cause of abortion and reproductive loss or what exactly the manifestations are of infection in pinnipeds [13,14].

Certain virulence factors are considered essential for the pathogenesis of *C. burnetii,* as this intracellular pathogen requires both seamless macrophage invasion and the ability to survive in the defensive host phagolysosome [17]. Two of the most important systems genetically encoded for the pathogenesis of *C. burnetii* are the lipopolysaccharide (LPS) and the genes affecting the type-4 secretory system (T4SS) [18]. The presence or absence of the genes encoding the O-antigen is used to distinguish between virulent and avirulent strains [18]. There is a considerable data deficiency with regard to the presence and absence of virulence factors in *C. burnetii* in terrestrial wildlife, and no published data for marine mammals. When considering the difficulty of studying this organism’s disease ecology and pathobiology using conventional microbiology and molecular methods, it becomes apparent that genome sequencing is essential to understanding these processes [1].

The main aims of this study were to use whole-genome sequencing to confirm the previous identification of *C. burnetii* in Australian fur seals and to gain insight into the phylogenetic relationship of this isolate to other pathogenic *C. burnetii* strains detected in terrestrial environments. The presence of other well-characterized markers of *Coxiella* diagnosis and genetic diversity were also examined alongside a preliminary investigation into the pathogenic potential of this genotype, as indicated by the presence of previously characterized *C. burnetii* virulence factors.

## 2. Materials and Methods

### 2.1. Whole-Genome Sequencing and Assembly

Genomic DNA was extracted from an Australian fur seal placental swab sample collected from Kanowna Island (39°15′ S, 146°30′ E) in 2021. The placenta was produced from a presumed full-term birth at the peak of the normal pupping season for the species. No clinical data are available for the dam or the pup. This specimen had previously tested positive for *C. burnetii* DNA and undergone molecular typing as previously described [14].

Following library construction, genome sequencing was performed using the Novaseq platform (Illumina Australia and New Zealand, Melbourne, Australia) by the Australian Genome Research Facility (https://www.agrf.org.au/, accessed on 18 July 2022). Raw sequencing data were uploaded to the Short Read Archive (PRJNA962036).

The Illumina 150 bp paired-end reads were filtered and trimmed with Trimgalore (Version 0.4.5, https://github.com/FelixKrueger/TrimGalore, accessed on 8 November 2022) to identify and remove sequencing adapters and low-quality stretches of bases. Kraken (Version 2.0.8) [19] and Bracken [20] were utilized for assessment of the composition of the microbial communities present in the placental DNA extract using the NCBI genome and nucleotide databases (downloaded 15 April 2021).

Quality-filtered *Coxiella* reads identified by Kraken were subsequently assembled using SPAdes v 3.15.0 [21]. Assembled contigs were functionally annotated using Prokka3 (https://github.com/tseemann/prokka, accessed on 8 November 2022) [22]. The assembly was assessed for completeness using BUSCO7 (Version 3) [23], based on the identification of universal single-copy orthologs and the bacteria_odb9.

### 2.2. Phylogenetic Analysis

A phylogenetic tree was constructed using 1131 core genes identified between the newly sequenced *Coxiella* PG457 genome and the publicly available genomes of 12 *C. burnetii* strains (Table S1). All genomes used in the phylogenetic analysis were re-annotated using Prokka3 to ensure consistent gene calling in all genomes. The core genes were identified using Panaroo [24] running in Strict mode with the sequence identity threshold set to 98% and the alignment option set to “core”. The core gene alignment generated by Panaroo was inputted into Iqtree [25] to infer a maximum-likelihood tree using the GTR+I+G model and with 1000 bootstrap replicates. The *C. burnetii*-only phylogenetic tree was rooted using the novel *Coxiella* PG457 sequence.

A second phylogenetic tree using the 12 *C. burnetii* genomes plus *Rickettsiella grylli* (Accession number: GCF_000168295.1) and the *Coxiella* endosymbiont of *Rhipicephalus microplus* (accession number: GCF_002930125.1) was generated using a concatenated alignment of seven housekeeping genes (total length of 32,302 bp) (Table S2). The tree was created with Iqtree (accessed on 31 January 2023) using the GTR+I+G model on the concatenated alignment and rooted using *R. grylli*. 

### 2.3. Comparative Analysis

The draft genome assembly of the novel *Coxiella* PG457 was examined for the presence of (i) *Coxiella* IS1111 transposase sequences, (ii) MLVA gene sequences, and (iii) previously described *Coxiella* virulence factors. IS1111 sequences were screened for by a combination of read mapping to the *C. burnetii* Nine Mile RSA439 (NZ_CP018005.1) genome, BLAST searching of the genome assembly, and by use of the IS1111 element search tool available in Coxbase (https://coxbase.q-gaps.de/webapp/analysis/is1111, accessed on 28 January 2023) [22]. MLVA screening utilized the Coxbase MLVA typing tool (https://coxbase.q-gaps.de/webapp/analysis/mlva, accessed on 28 January 2023). BLAST was used to determine the presence of specific *Coxiella* virulence factors in the *Coxiella* PG457 draft genome assembly.

## 3. Results

### 3.1. Description of Novel Australia Coxiella Draft Genome Assembly

The properties of the assembled draft genome for *Coxiella* PG457 are presented in Table 1. De novo assembly of the *Coxiella*-like quality filtered reads resulted in a genome scaffold of 1.9 MB comprising 204 contigs. Automated annotation resulted in 2327 predicted coding sequences (CDS). The assembled *Coxiella* PG457 draft genome had a G + C content of 42% with three rRNA operons and 43 tRNAs. BUSCO analysis of the *C. burnetii* PG457 draft genome’s completeness revealed a score of 92.6% [1].

To confirm the presumptive identity of the *Coxiella*-positive sample sequenced, the full-length 16S rRNA sequence was extracted from the assembled *Coxiella* draft genome and compared with available sequences in the NCBI database using nucleotide BLAST. This analysis revealed 99.7% identity to the full-length 16S rRNA sequences of a range of *C. burnetii* strains, including the *C. burnetii* Nine Mile RSA439 reference genome (CP035112.1), thus identifying this *Coxiella* strain as belonging to the species *C. burnetii*. Henceforth, this strain is referred to as *C. burnetii* PG457. Additionally, PG457 was analyzed with JSpeciesWS (https://jspecies.ribohost.com/jspeciesws/, accessed on 5 May 2023) for a pairwise comparison (ANIbMA and ANImMA) and Tetra correlation search to two reference strains of *C. burnetii* to ensure the 16s rRNA nucleotide BLAST did not produce erroneous results (Table S3).

An all-versus-all BLAST comparison of *C. burnetii* PG457 against the completed genome of *C. burnetii* Nine Mile RSA439 also demonstrates that PG457 is assembled with a high level of completeness and synteny (Supplementary Figure S1). *C. burnetii* PG457 shares > 90% sequence identity, and limited evidence for genome rearrangement and unique genomic regions, compared with the *C. burnetii* reference strain.

### 3.2. Phylogenetic Analysis of C. burnetii PG457

**Figure 1:**
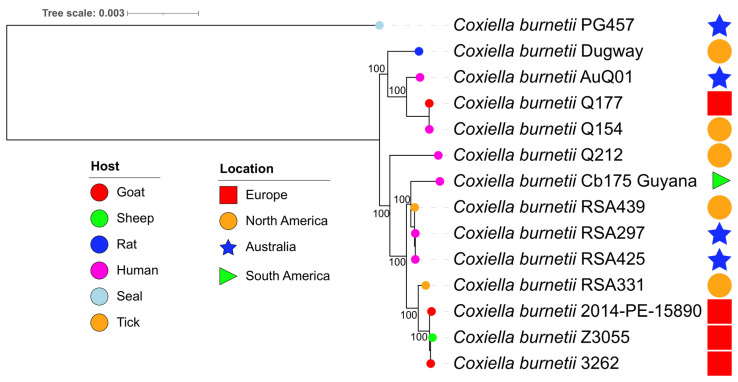
Rooted phylogenetic tree of 13 *C. burnetii* isolates, including *C. burnetii* PG457, overlaid with host and isolate locations. The phylogenetic tree was constructed based on alignment of core 1131 genes identified using Panaroo [24], and bootstrap values are shown as percentages of 1000 replicants. Scale indicates number of substitutions per site. There is a possibility that *C. burnetii* strains RSA425 and RSA297, while originally Australian isolates, may have been contaminated with, and overgrown by, North American strains during passaging in laboratories many decades ago (Personal communication S. Graves).

To understand the phylogenetic relationships of the novel *C. burnetii* strain sequenced in this study to other *C. burnetii* strains isolated in Australia, Europe, and the North America from human and animal hosts, a phylogenetic tree was constructed using an alignment of 1131 concatenated core genes extracted from 12 publicly available *C. burnetii* genomes (Figure 1). This analysis revealed that *C. burnetii* PG457 clustered away from other sequenced *C. burnetii* strains, forming its own distinct lineage from terrestrial *C. burnetii* strains isolated in Australia as well as those from the rest of the world.

**Figure 2:**
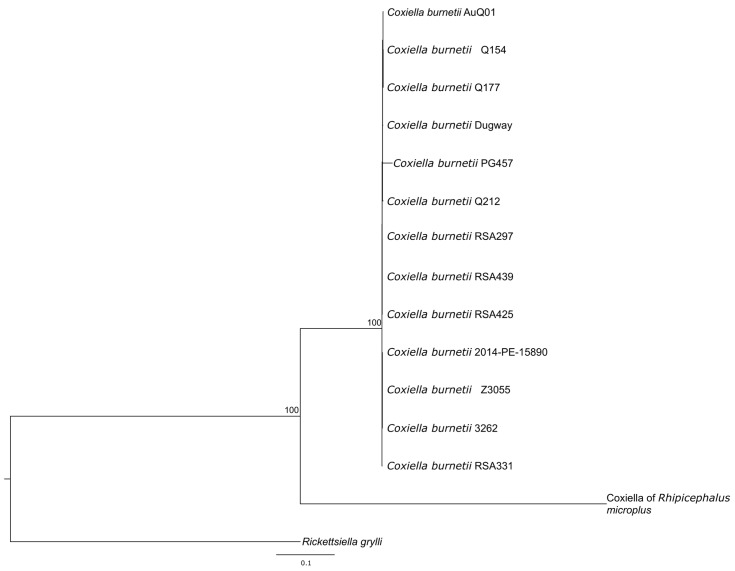
Maximum likelihood phylogenetic tree based on the concatenated alignment of 7 conserved genes (32,301 bp) detected in the genomes of 13 *C. burnetii* strains (including *C. burnetii* PG457) and a *Coxiella*-like endosymbiont/bacterium (*Coxiella* of *Rhipicephalus microplus*). The tree was rooted to *Ricketsiella grylli*. Scale indicates number of substitutions per site.

To gain a broader understanding of these genetic differences, we expanded the phylogenetic analysis to include a *Coxiella*-like organism, a *Coxiella* endosymbiont of *Rhipicephalus microplus*. A phylogenetic tree constructed using a concatenated alignment of seven *Coxiella* housekeeping genes revealed that, relative to *Coxiella*-like organisms, the *C. burnetii* PG457 strain is distinguishable but nevertheless is closely related to other well-characterized *C. burnetii* strains (Figure 2).

### 3.3. Identification of Previously Described C. burnetii Features in the Genome of C. burnetii PG457

Having established the identity of the *C. burnetii* isolate, preliminary genomic analysis was performed to detect the presence of common molecular marker genes and/or markers of *C. burnetii* pathogenicity.

#### 3.3.1. Coxiella IS1111 Insertion Sequence

The IS1111 transposase sequence is a multicopy genetic element widely targeted for the PCR detection and differentiation of *C. burnetii* strains [26]. To detect IS1111 sequences in the *C. burnetii* PG457 draft genome assembly, *C. burnetii* PG457 reads were mapped to the *C. burnetii* Nine Mile RSA439 genome (NZ_CP018005.1). No reads were found to align with any of the 20 IS1111 transposase sequences present in the *C. burnetii* reference genome [1]. BLAST searching of the *C. burnetii* PG457 genome with IS1111 sequences also failed to detect any of this particular transposase sequence. As a final search strategy, the *C. burnetii* PG457 genome assembly was imported into the IS1111 element search tool (https://coxbase.q-gaps.de/webapp/analysis/is1111, accessed on 28 January 2023) [27]. No IS1111 sequences were identified. Together, these results suggest that the IS1111 transposase is absent from the *C. burnetii* PG457 genome.

#### 3.3.2. C. burnetii MLVA Gene Targets

The *C. burnetii* MLVA typing scheme, consisting of a panel of 17 MLVA loci, is one of many used for fine-detailed genetic differentiation of *C. burnetii* strains [28]. To identify the presence of MLVA loci, the *C. burnetii* PG457 draft genome assembly was inputted into the Coxbase MLVA typing tool (https://coxbase.q-gaps.de/webapp/analysis/mlva, accessed on 28 January 2023). Compared with the *C. burnetii* Nine Mile RSA439 genome, only five loci (ms26, ms27, ms28, ms30, and ms31) could be detected in the *C. burnetii* PG457 draft genome assembly (Table 2).

#### 3.3.3. Putative C. burnetii Virulence Factors

To gain a preliminary insight into the pathogenesis of this marine *C. burnetii* strain, we examined the *C. burnetii* PG457 draft genome assembly for the presence of previously characterized *C. burnetii* virulence factors. This included searches for components of systems used to interact with the host cell during a *C. burnetii* infection as well as virulence factors that can be used to differentiate pathogenic and nonpathogenic *C. burnetii* strains (Table 3). All but one of the genes (*adaA*) searched for could be found in the *C. burnetii* PG457 draft genome assembly, including genes associated with the synthesis of *Coxiella* O-antigen, a component of the *C. burnetii* lipopolysaccharide, as well as structural components and effectors of the T4SS involved in manipulation and survival in the host cell. This suggests that *C. burnetii* PG457 is likely to be virulent, both for Australian fur seals and for terrestrial mammals, including humans.

## 4. Discussion

*C. burnetii* infections in wildlife have been speculated to be a conservation risk while also posing a threat of spillover infections to humans and domesticated animals [7]. In the current study, we sequenced the genome of a *C. burnetii* strain detected in an Australian fur seal to gain insight into its relationship with terrestrial *C. burnetii* strains but also to begin to understand the potential for this pathogen to cause disease in wildlife hosts.

Preliminary phylogenetic analysis of the core genes identified in the whole genome assembly suggests that this previously reported [9,14,35] bacterium is a strain of *C. burnetii*. However, it is genetically distinct from other pathogenic *C. burnetii* strains detected in humans and other terrestrial animals both in Australia and elsewhere. While genetically distinct from those strains, the genome of *C. burnetii* PG457 contains all of the basic genetic hallmarks of previously described *C. burnetii* genomes, including a similar estimated (i) genome size (1.9–2.1 Mb range) and G + C content (42.4–42.9%) [36] and (ii) number of stable rRNA and tRNA genes. Unfortunately, the sequenced genome of PG457 is not circular. The BUSCO analysis revealed that 7.4% of the sequenced genome is fragmented or missing. It is unknown to what extent this would affect downstream analysis. It is conventionally assumed that sequenced genomes need to have a minimum N50 of 1Mb to ensure no errors occur in the annotation, allowing for conserved genome synteny [37], which the assembled genome of PG457 meets and exceeds.

There are exceptionally few reports of *C. burnetii* lacking the IS1111 transposase, including only one in humans [35], and sequencing data are not available. Sequencing of *C. burnetii* strain PG457, which lacks IS1111, illustrates the potential evolutionary relationships between some of the *Coxiella*-like endosymbionts that lack this insertion sequence and *C. burnetii*. This is the first sequencing of a *C. burnetii* that lacks the IS1111 gene. Previous marine mammal studies reporting poor or absent amplification of the IS1111 may very well have been reporting on the same genotype as in this current study [13,14]. This insertion sequence is a multicopy and is often used to differentiate isolates from one another [26]. The genome sequence of this current isolate further supports the hypothesis that the IS1111 transposase is absent from marine mammal strains. The apparent absence of the IS1111 could relate to the missing MLVA loci ms23 and ms33, as these are loci that notoriously contain recognition sites for IS1111 [38,39].

A very distinct MLVA signature appears to be present in this novel isolate. The use of MLVA is considered highly discriminatory between different *C. burnetii* genotypes [40]. Typical Australian genotypes of *C. burnetii* have the presence of IS1111, and additionally, three critical MLVA loci, namely ms24, ms28, and ms33 [15]. Previous studies of *C. burnetii* in Australian fur seals have shown strong evidence that only ms28 is present in these isolates [14,41]. Based on the MLVA-associated genomic grouping, this novel isolate appears to cluster outside of any of the currently accepted genomic groups and any of those represented in Australia [38]. We propose that an MLVA signature for marine mammal isolates of *C. burnetii* will consist of ms26, ms27, ms28, ms30, and ms31. This assumption, made from the currently available genome sequence data, needs to be verified in samples from other marine mammals and in silico.

Although the exact pathogenic manifestations of *C. burnetii* in marine mammals are still to be fully illustrated, it is apparent from the initial sequencing data of *C. burnetii* in Australian fur seals that certain key virulence factors appear to be present in the genome that are shared with pathogenic terrestrial genotypes. Conventionally, genomic groups I and IV contain the most pathogenic genotypes of *C. burnetii* [38], but the inability to group this novel isolate into any of these previously described genomic groups makes it more difficult to infer pathogenicity. Ideally, the use of a guinea pig model might better illustrate the true pathogenic ability of this marine mammal isolate because it is considered one of the gold standard models to infer pathogenicity [42]. In human cases of Q fever, it appears that certain SNP (single nucleotide polymorphism) genotypes are more likely to cause disease [17], but this has not been determined for PG457. The presence of the tadA, O-antigen, ompA, mceA, dotA, and dotB genes at 94.6–97.6% with the Nine-Mile strain would suggest a strong presence of the LPS and T4SS [18]. This potentially indicates the ability of this novel isolate to induce significant pathology in marine mammals and probably pose a zoonotic risk to persons working with these animals.

Ideally, this novel genome sequence needs to be compared with those of northern-hemisphere marine mammals to determine the evolutionary relatedness of these various strains. Further surveillance is needed to determine if any horizontal transmission can occur between marine mammals and terrestrial mammals. If it is possible to culture *C. burnetii* from Australian fur seals, potentially a full genome could be sequenced and a viable inoculum made available, to determine its virulence in guinea pigs. If virulent, it is possible that this bacterium has been playing a role in declining marine mammal populations.

## Figures and Tables

**Table 1:** *Coxiella* PG457 draft genome assembly statistics.

Criteria	Statistic
Number of scaffolds	201
Number of contigs	204
Scaffold sequence length	1.907 MB
Maximum scaffold length	115,883 KB
Maximum contig length	105,116 KB
Percentage of sequence in scaffold > 50	60.15%

**Table 2:** Detection of *C. burnetii* MLVA loci in the *C. burnetii* PG457 and *C. burnetii* Nine Mile RSA439 genome using the Coxbase MLVA search tool (https://coxbase.q-gaps.de/webapp/analysis/mlva).

Strain	Locus Characteristic	MS01	MS03	MS20	MS21	MS22	MS23	MS24	MS26	MS27	MS28	MS30	MS31	MS33	MS34
*C. burnetii* PG457	Product length	N.D. ^a^	N.D.	N.D.	N.D.	N.D.	N.D.	N.D.	132	263	132	306	142	N.D.	N.D.
	Flank length	N.D.	N.D.	N.D.	N.D.	N.D.	N.D.	N.D.	104	249	112	205	106	N.D.	N.D.
	Repeat size	N.D.	N.D.	N.D.	N.D.	N.D.	N.D.	N.D.	9	6	6	18	7	N.D.	N.D.
	Repeat number	N.D.	N.D.	N.D.	N.D.	N.D.	N.D.	N.D.	3	2	3	5	5	N.D.	N.D.
*C. burnetii* RSA439	Product length	248	227	402	210	246	157	344	150	276	150	306	150	262	210
	Flank length	176	142	96	136	174	90	135	104	249	112	205	106	193	175
	Repeat size	16	12	33	12	11	7	7	9	6	6	18	7	7	6
	Repeat number	4	7	9	6	6	9	29	5	4	6	5	6	9	5

^a^ N.D. = Not detected.

**Table 3:** Preliminary analysis of the previously described *Coxiella* virulence factors in the *C. burnetii* PG457 draft genome assembly.

Gene/Gene Product	Product Description and Function	Detected in *C. burnetii* PG457?	Nucleotide Identity to *C. burnetii* RSA439 Gene	Percentage Gene Coverage	Nucleotide Identity to *C. burnetii* Cb175 Gene	Percentage Gene Coverage
*tadA*	Type 2 and Type 4 family secretion system ATPase, TadA; predicted involvement in *Coxiella* adherence [29]	Yes	96.2%	99%	96.2%	100%
O-antigen/LPS	O-antigen ligase family protein, membrane transport (lptA,B,C); synthesize components of the *Coxiella* lipopolysaccharide unique to virulent strains [30]	Yes	95.4%	100%	95.43%	100%
*ompA*	Outer Membrane Protein A; *Coxiella* invasin [31]	Yes	96.8%	100%	96.8%	100%
*mceA*	Mitochondrial *Coxiella* effector protein A; effector targeting host cell mitochondria during *Coxiella* infection [32]	Yes	97.6%	100%	97.6%	100%
*dotA*	Type IV secretion system protein, DotA; structural component of the Dot/Icm Type IV secretion system complex [33]	Yes	94.6%	100%	94.6%	100%
*dotB*	Type IV secretion system protein, DotB; structural component of the Dot/Icm Type IV secretion system complex [33]	Yes	96.6%	99%	96.57%	99%
*adaA*	*Coxiella burnetii*-specific acute disease antigen, adaA; outer membrane protein, originally thought to differentiate strains causing acute and chronic Q fever [34]	No	−	−	−	−

## Data Availability

All data not supplied as Supplementary Files are available on request.

## Supplementary Materials

The following supporting information can be downloaded at: https://www.mdpi.com/article/10.3390/pathogens12070893/s1. Figure S1: Whole genome alignment of the Coxiella burnetii PG457 (bottom) and C. burnetii Nine Mile RSA439 reference genome (top). Red shading indicates a nucleotide identity of > 90%; e Phylogenetic tree was constructed using 1131 core genes identified between the newly sequenced Coxiella PG457 genome and the publicly available genomes of 12 C. burnetii strains; e Seven conserved “housekeeping” genes (32,301 bp) detected in the genomes of 13 C. burnetii strains (including C. burnetii PG457) and a Coxiella-like organism (Coxiella of Rhipicephalus microplus); Table S1: Coxiella burnetii sequenced genomes from NCBI; Table S2: Coxiella burnetii conserved house-keeping genes; Table S3: C. burnetii PG457 ANIbMA, ANIMMA and TETRA analysis.
