# Supplementary figures and images for: A Novel Marine Mammal Coxiella burnetii—Genome Sequencing Identifies a New Genotype with Potential Virulence
Source: Pathogens. 2023 Jun 29;12(7):893. doi: 10.3390/pathogens12070893 (PMC10386718; doi:10.3390/pathogens12070893)

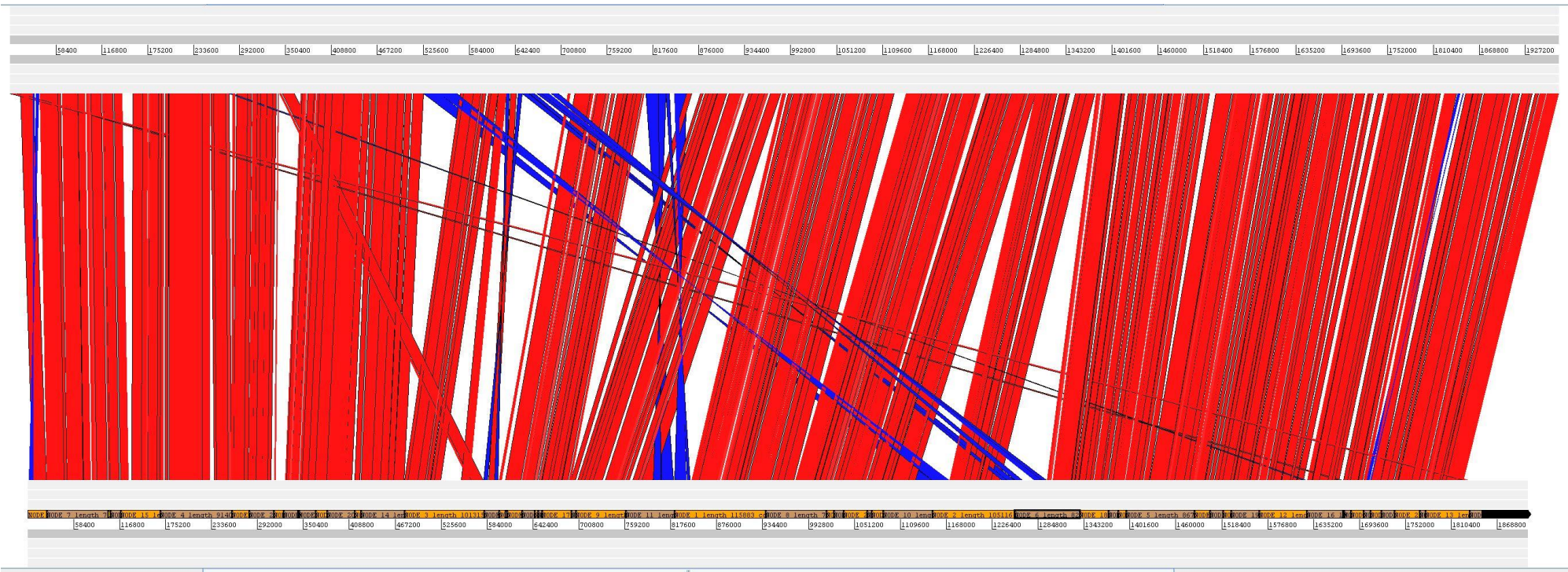

Supplement: Supplementary file 1 [file pathogens-12-00893-s001.zip › Figure S1.pdf]
